# VIRGINIA BELL: AN ORAL HISTORY OF A GERONTOLOGICAL SOCIAL WORKER AND PIONEER IN DEMENTIA CARE

**DOI:** 10.1093/geroni/igad104.2184

**Published:** 2023-12-21

**Authors:** Natalie Pope, Mohammad Hossain, Allison Gibson

**Affiliations:** University of Kentucky, Lexington, Kentucky, United States; University of Kentucky, Lexington, Kentucky, United States; Saint Louis University, St. Louis, Missouri, United States

## Abstract

This presentation highlights the contributions of a gerontological social worker and dementia expert who developed an innovative model of dementia care known as the Best FriendsTM model. Virginia’s story is one of a second-chapter career. She spent most of her adult life raising her five children and supporting her husband, who worked in ministry. At age 62, she graduated with her Master of Social Work and completed her practicum at a newly established center on dementia research (the Sanders-Brown Center on Aging at the University of Kentucky). While this practice experience contributed to her professional trajectory as a gerontological social worker, Virginia draws most heavily on lessons learned from her relationships. Her intersecting identities as a daughter, wife, mother, and grandmother informed her pioneering work in dementia care by teaching her the value of mutuality, hard work, and community. Through several in-depth interviews, this oral history project creates a space for conversation across generations –dialogues between two women, both social workers and gerontologists. The narrator, age 98, reflects on the people and experiences that shaped her approach to caring for people with dementia from a relational-centered approach. The interviewer (age 42) assumes a co-interpreter role with Virginia, documenting what shaped her thinking about dementia. This presentation highlights 1) how oral history can be used to document the experiences and knowledge of social service innovators and 2) Virginia Bell’s unique contribution via the Best Friends model.

